# From Impaction to Eruption, Simplified Management of Two Teeth Impacted by a Large Mandibular Cyst: A Clinical Case Report

**DOI:** 10.3390/dj14090614

**Published:** 2026-09-21

**Authors:** Ioannis P. Zogakis, Chrysanthi Anagnostou, Theodoros Lillis

**Affiliations:** 1 Department of Orthodontics, School of Dentistry, Faculty of Health Sciences, Aristotle University of Thessaloniki, 54124 Thessaloniki, Greece; 2 Department of Hygiene, Social-Preventive Medicine and Medical Statistics, School of Medicine, Faculty of Health Sciences, Aristotle University of Thessaloniki, 54124 Thessaloniki, Greece; 3 Department of Oral Surgery, Implantology and Radiology, School of Dentistry, Faculty of Health Sciences, Aristotle University of Thessaloniki, 54124 Thessaloniki, Greece

**Keywords:** impacted teeth, tooth impaction, mandibular cyst, dentigerous cyst, interdisciplinary treatment

## Abstract

**Background/Objectives**: Dentigerous cysts are odontogenic cysts enclosing the crown of an unerupted, developing tooth, therefore disrupting the normal eruption process, leading to tooth impaction. This case report describes the successful multidisciplinary management of a large mandibular clinicoradiographic dentigerous cyst associated with two impacted permanent teeth, highlighting the potential for spontaneous tooth eruption following conservative surgical treatment. **Methods**: A healthy 14-year-old male presented with a large mandibular cyst associated with two unerupted permanent teeth. Clinical examination revealed mixed, spaced dentition with bilateral Class I molar relationships. Radiographic examination demonstrated a well-defined radiolucent lesion in the mandibular body surrounding the crown of an impacted first premolar and displacing the adjacent permanent canine. As no histopathological examination was performed, the diagnosis was established on clinical and radiographic grounds. Treatment objectives were to resolve the cystic lesion, promote spontaneous eruption of the impacted teeth, achieve alveolar bone regeneration and resolve the associated malocclusion. Marsupialization was performed, followed by periodic clinical and radiographic monitoring. Active orthodontic treatment was initiated 29 months after surgery, once satisfactory spontaneous tooth eruption and bone healing had been achieved. **Results**: Marsupialization resulted in resolution of the cyst, spontaneous eruption of both impacted permanent teeth, substantial alveolar bone regeneration with restoration of normal trabecular architecture and interproximal bone height, and successful subsequent orthodontic correction of the malocclusion. **Conclusions**: Conservative management with marsupialization can effectively treat large clinicoradiographic dentigerous cysts in growing patients while preserving the associated teeth and promoting spontaneous eruption and bone regeneration. Careful long-term follow-up and appropriately timed orthodontic intervention can result in favorable functional and radiographic outcomes, supporting a biologically driven, minimally invasive treatment approach, supported by appropriately timed technical interventions.

## 1. Introduction and Clinical Significance

Dentigerous cysts are benign odontogenic lesions that typically remain asymptomatic and are discovered incidentally. Inflammatory stimuli from a periapical granuloma of a non-vital deciduous tooth may contribute to their pathogenesis [1]. Dentigerous cysts originate within the confined space between the inner and outer enamel epithelia of the dental follicle of an unerupted tooth and represent approximately 17–18% of jaw cysts, thus are considered the second most common type of odontogenic cyst after radicular cysts [2,3].

Clinically, these cysts are generally slow-growing and asymptomatic, often presenting as a painless swelling. As they expand, they typically enclose the crown, which projects into the lesion and is directed towards its center [1]. As they enlarge, they may lead to displacement of adjacent teeth, as well as resorption of dental structures and surrounding bone. In cases of extensive enlargement, patients may also experience pain or discomfort [4].

Radiographically, they present as well-defined unilocular radiolucencies, with their boundaries commonly attached at the cemento–enamel junction [1,5]. Larger lesions may produce expansion of the cortical bone. Although uncommon, some dentigerous cysts may exhibit a multilocular appearance on panoramic radiographs, a feature thought to result from cystic expansion through regions of varying bone density. In such cases, differentiation from more aggressive odontogenic lesions, including ameloblastoma, odontogenic keratocyst and other odontogenic tumours, is essential [4,6].

Therapeutic strategies remain a topic of debate, with management options ranging from complete enucleation and extraction of the associated tooth [7,8] to more conservative approaches such as marsupialization or decompression, aimed at preserving the involved dentition [9,10,11,12]. If dentigerous cysts are not managed appropriately, they exhibit a marked propensity for progressive enlargement, often resulting in significant resorption of adjacent osseous structures and potentially causing asymmetrical mandibular expansion and facial deformity [8].

The purpose of this article is to present the case of a young individual with a large clinicoradiographically diagnosed mandibular dentigerous cyst, associated with two unerupted permanent teeth that erupted spontaneously after surgical treatment. The principal treatment objectives included the resolution of the pathologic entity, the spontaneous eruption of the impacted teeth, the regeneration of the alveolar bone and the resolution of the malocclusion.

## 2. Case Presentation

### 2.1. Case History

A healthy 14-year-old male patient was referred to the orthodontic clinic by a general dentist for evaluation for space maintenance, following extraction of a non-vital primary molar in the mandible. Upon extraoral inspection, it was observed that the patient’s face was well-balanced and relatively symmetrical, with a straight profile. The intraoral clinical examination revealed a mixed, spaced dentition and Class I molar relations bilaterally and a 3 mm deviation of the mandibular dental midline to the right. The patient was asymptomatic, with no history of pain, swelling, infection, altered sensation, or facial asymmetry. The adjacent permanent teeth showed normal clinical appearance, positive responses to cold vitality testing and electric pulp testing, and healthy periodontal conditions, with no evidence of increased mobility or periodontal abnormalities.

**Figure 1:**
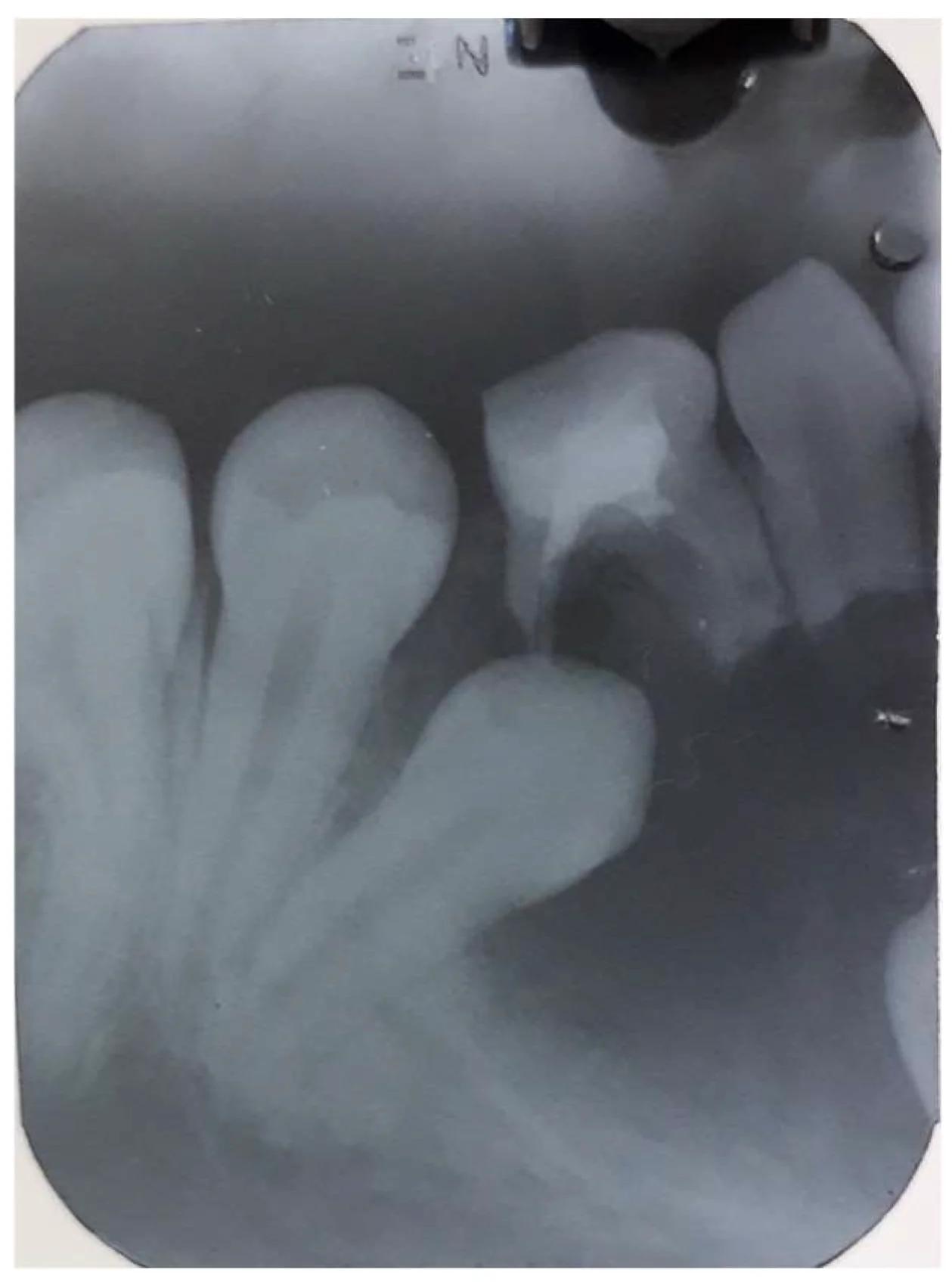
Pretreatment periapical radiograph.

**Figure 2:**
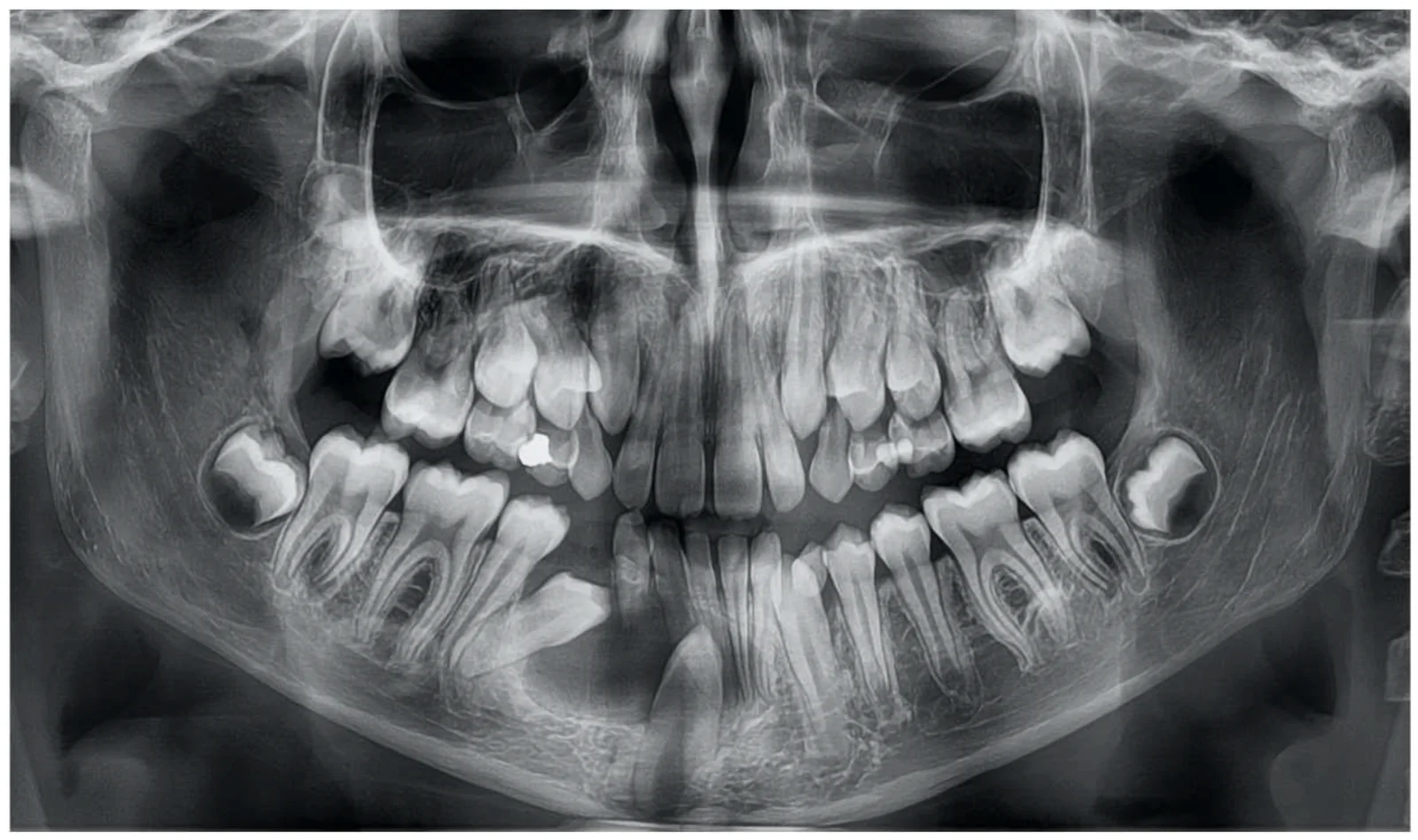
Initial panoramic radiograph.

**Figure 3:**
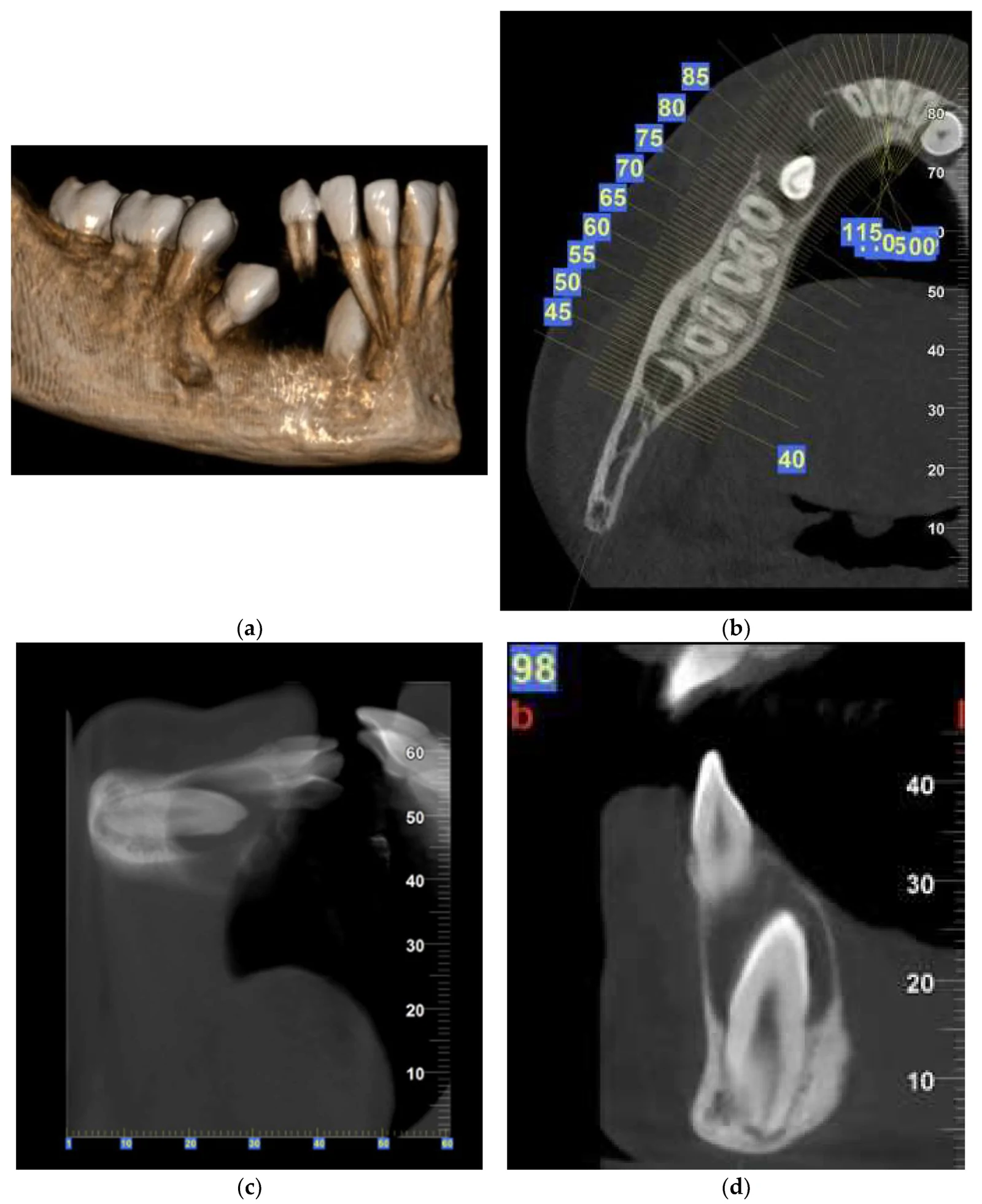
Initial CBCT assessment of the mandibular lesion: (**a**) three-dimensional reconstruction demonstrating the overall extent of the lesion and relationship with the impacted teeth; (**b**–**d**) multiplanar CBCT sections showing the lesion dimensions and anatomical relationships. The cyst measured approximately 30 × 25 × 20 mm (AP × SI × BL).

The periapical radiograph provided by the general dentist revealed the presence of a cystic lesion between the apex of a non-vital primary molar and the crown of its successor premolar (Figure 1). Therefore, the patient was referred for further diagnostic imaging, including a panoramic radiograph (Figure 2) and a localized cone–beam computed tomography (CBCT) (Figure 3). Radiographic examination revealed a large, well defined radiolucent lesion in the body of the mandible, surrounding the crown of an unerupted first premolar and displacing the adjacent permanent canine, with severe bone loss, as well as the presence of two impacted, permanent teeth, the right mandibular canine and the first premolar. CBCT examination demonstrated a well-defined radiolucent lesion in the right mandibular body, surrounding the crown of the unerupted mandibular right first premolar (tooth 44) and extending toward the unerupted mandibular right canine (tooth 43). The lesion was associated with displacement of the mandibular right canine and alteration of the normal axial inclination of the involved permanent teeth. The CBCT images demonstrated buccal and lingual cortical expansion and thinning, without evidence of cortical perforation. The relationship between the lesion and the inferior alveolar canal was assessed, with no signs of direct canal involvement. The roots of adjacent permanent teeth showed no evidence of resorption.

The impacted mandibular right canine and first premolar demonstrated incomplete root development. Both teeth exhibited altered angulation and ectopic eruption positions. The available space within the dental arch were considered adequate, suggesting potential for spontaneous eruption following conservative management.

Radiographic findings were consistent with a dentigerous cyst surrounding the crown of the unerupted first premolar and displacing the adjacent permanent canine, with associated bone loss. The differential diagnosis included other odontogenic cystic lesions and developmental odontogenic lesions, such as odontogenic keratocyst and unicystic ameloblastoma; however, the clinical and radiographic characteristics favored a dentigerous cyst.

### 2.2. Treatment Considerations and Management of the Case

The standard management of dentigerous cysts typically involves enucleation of the cyst along with extraction of the associated tooth. In cases of extensive lesions, where enucleation poses a significant risk of damaging vital anatomical structures or when tooth preservation is desired, alternative surgical approaches such as decompression or marsupialization are considered. Beyond these clinical considerations, the selection of a specific clinical approach is largely influenced by the clinician’s experience, training and preferences [13]. In our case, where the cyst affected an adolescent patient and was associated with developing teeth, our treatment goal was to facilitate the spontaneous or orthodontically guided eruption of the impacted teeth into a functional position within the dental arch. Therefore, marsupialization was our first-line treatment due to its conservative, minimally invasive nature, promoting lesion regression and healing with minimal risk to adjacent anatomical structures, while also supporting the natural eruption of the affected teeth [14,15]. Although certain limitations exist, such as the prolonged healing period, including the requirement for frequent follow-up visits and radiographic monitoring, and the importance of patient and parental cooperation for a successful outcome, the preservation of the involved teeth, thus maintaining a complete dental arch, and the potential for spontaneous eruption without the necessity for subsequent orthodontic intervention outweigh the associated disadvantages.

### 2.3. Treatment Progress—Surgical Management

**Figure 4:**
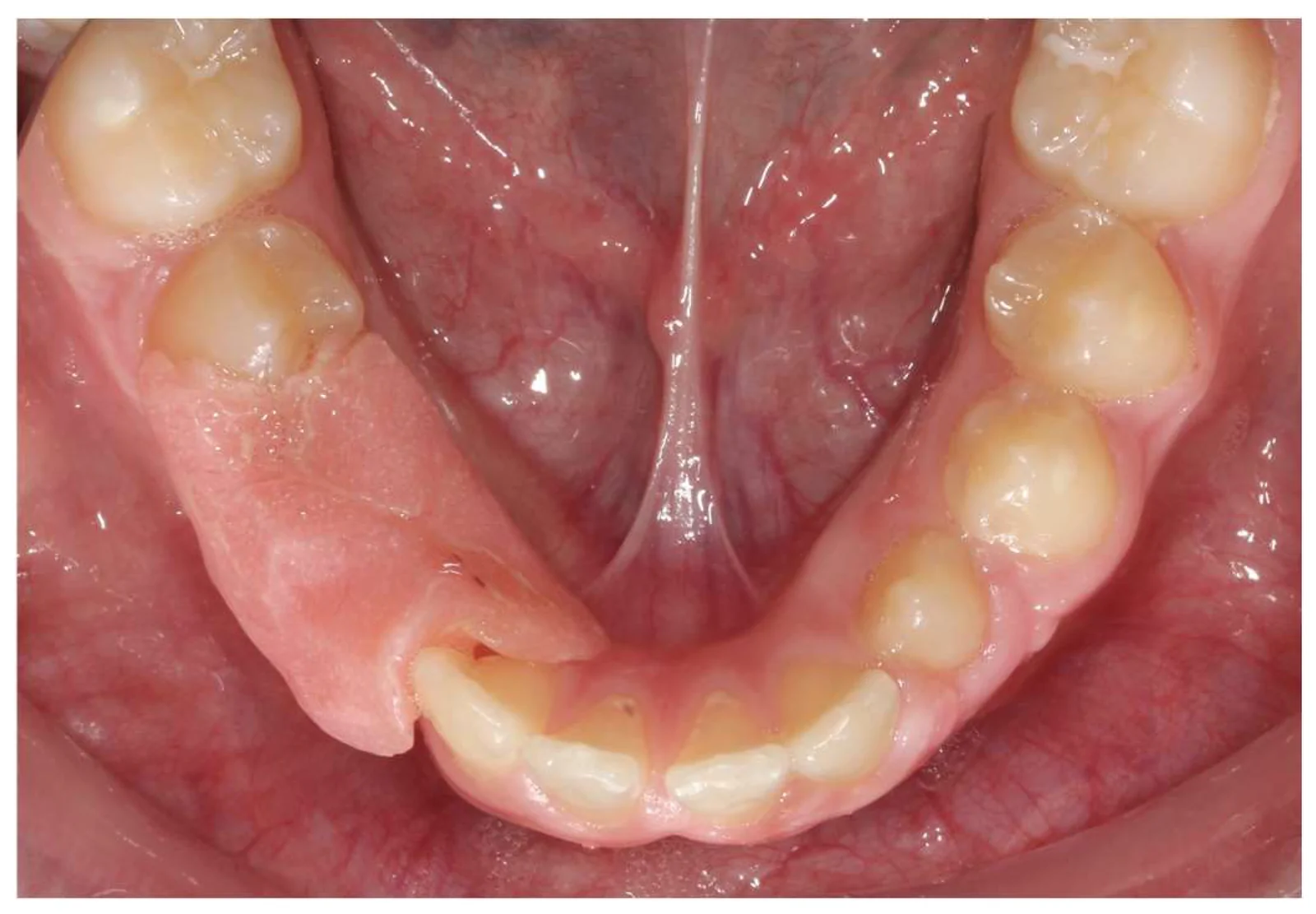
The placement of the acrylic obturator allowing for effective cyst drainage.

**Figure 5:**
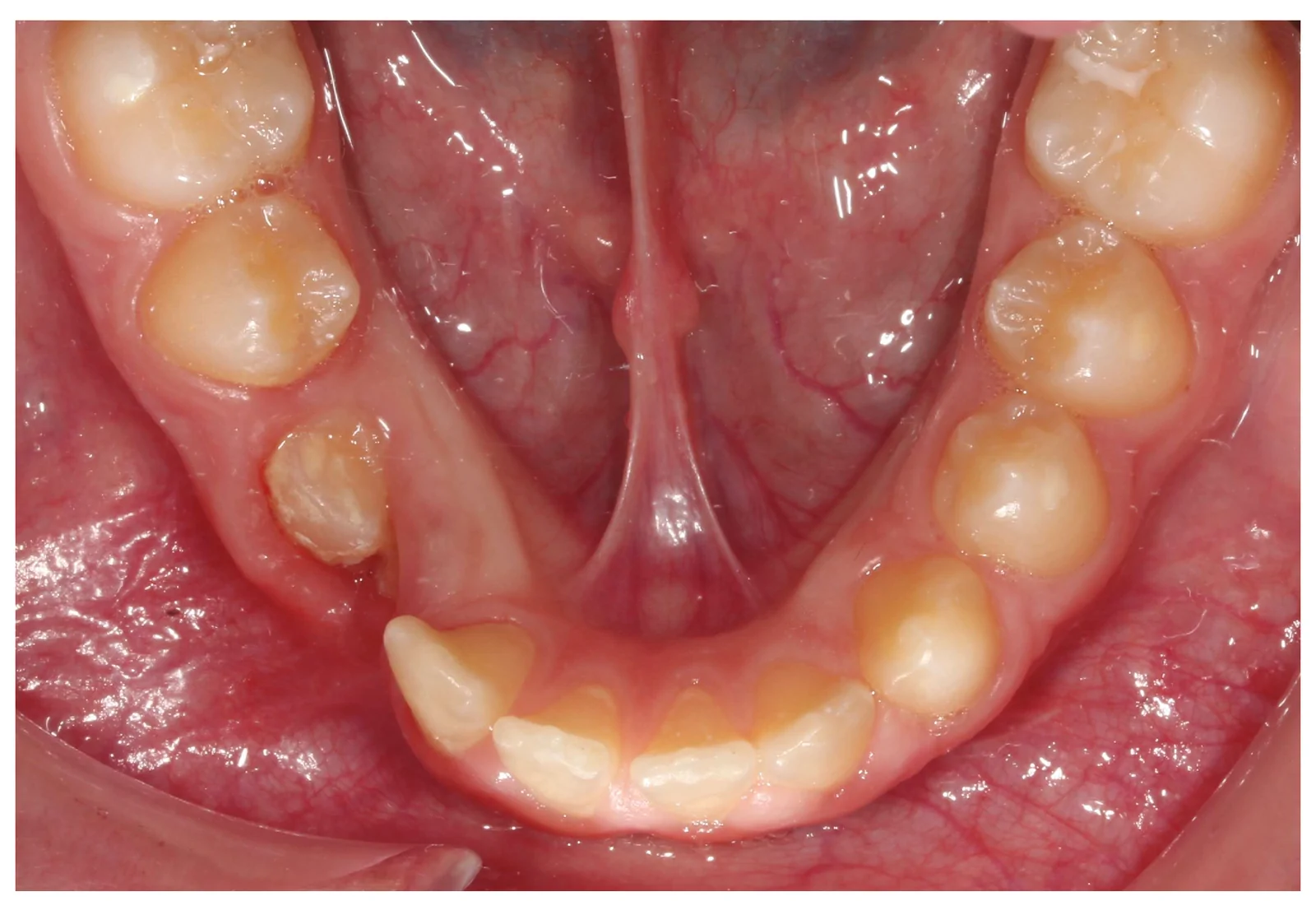
Clinical intraoral photograph obtained 7 months after marsupialization demonstrating the spontaneous eruption of the mandibular right first premolar during the healing phase.

**Figure 6:**
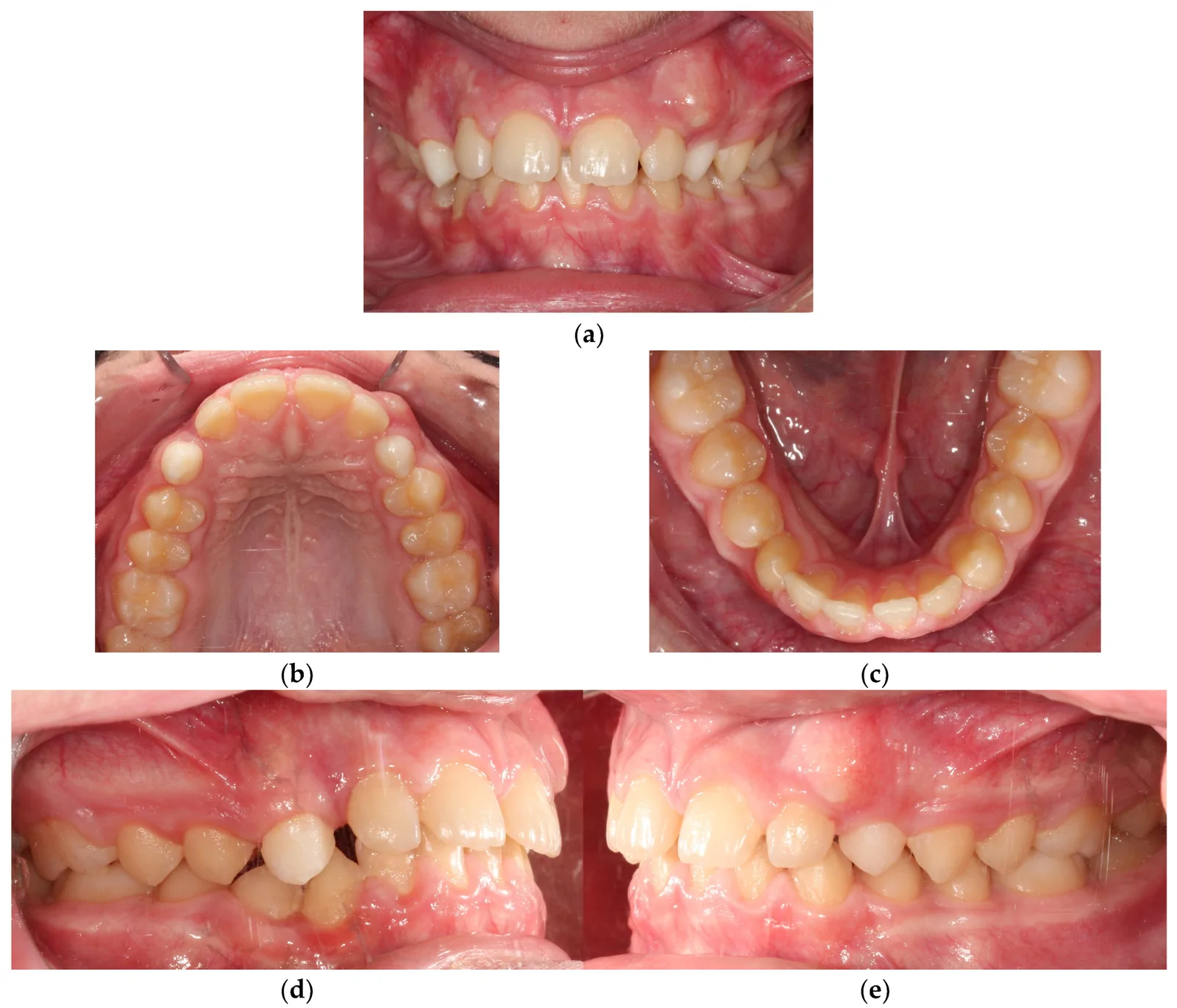
Pretreatment intraoral photographs obtained at the completion of the marsupialization phase: (**a**) frontal view, (**b**) maxillary occlusal view, (**c**) mandibular occlusal view, (**d**) right lateral view, and (**e**) left lateral view. The photographs demonstrate spontaneous eruption of the previously impacted mandibular right canine and first premolar. Before initiation of active orthodontic treatment, the maxillary primary canines had exfoliated, and a period of observation was maintained to allow satisfactory spontaneous eruption of the permanent maxillary canines before appliance placement.

The surgical treatment included marsupialization of the cyst. The surgical procedure was carried out by an oral surgeon under local anesthesia. Preoperative aspiration was performed, yielding a clear, straw-colored fluid, consistent with a cystic lesion. No purulent material or hemorrhagic fluid was obtained and there were no clinical findings suggestive of secondary infection. Cyst drainage was performed via the extraction socket of the deciduous molar, which provided unobstructed access to the cystic cavity, and a portion of the cystic wall was excised. The epithelial lining of the cyst was then sutured in continuity with the adjacent oral mucosa. Histopathological examination was not performed because, owing to the extreme thinness and friability of the cystic lining at the incision site, obtaining a viable and representative tissue specimen was technically challenging. Although histopathological confirmation was therefore unavailable, the lesion demonstrated an excellent clinical course during follow-up, characterized by rapid bone healing and successful orthodontic alignment, with no clinical or radiographic findings suggestive of recurrence or aggressive behavior. These favorable outcomes retrospectively supported the benign clinical nature of the lesion. An acrylic obturator, formed by an alginate impression, was placed, enabling continuous draining and regular patient-performed irrigations with normal saline twice daily (Figure 4). The obturator was maintained during the initial healing period and was periodically adjusted according to changes in the surgical site and eruption progress to prevent obstruction of the drainage pathway and avoid interference with bone healing and tooth eruption. The patient was followed regularly at the surgical dental clinic, with clinical evaluations and radiographic monitoring performed throughout the healing period. The patient demonstrated good compliance with the irrigation protocol, and no treatment-related complications were observed. Seven months after marsupialization, clinical examination demonstrated favorable spontaneous eruption of the mandibular right first premolar, reflecting the progressive recovery of the eruption pathway following conservative surgical management (Figure 5). Marsupialization was considered complete when the lesion showed clinical resolution, sustained reduction of the radiolucent area on serial radiographs, evidence of progressive bone healing, and favorable spontaneous eruption of the associated impacted teeth, allowing transition to the orthodontic phase of treatment. Pretreatment intraoral photographs obtained before appliance placement are presented in Figure 6.

No tissue specimen was submitted for histopathological examination; therefore, the diagnosis was based on the characteristic clinical and radiographic features. The patient was maintained under continuous radiographic surveillance by the orthodontist, with sequential imaging obtained to monitor the eruption of the impacted teeth and the progressive reduction of the lesion with associated osseous regeneration. Although the favorable radiographic evolution supported the conservative management approach and the clinical outcome achieved, it cannot replace histopathological examination or definitively exclude other odontogenic lesions. The observed radiographic changes were not suggestive of an aggressive or neoplastic process; however, histopathological examination remains necessary for definitive diagnosis.

### 2.4. Treatment Progress—Orthodontic Management

The orthodontic problem list included mixed dentition with generalized spacing, delayed eruption and impaction of the mandibular right canine and first premolar and an altered eruption pattern secondary to the cystic lesion. The patient presented with bilateral Class I molar relationships and a 3 mm deviation of the mandibular dental midline to the right, requiring correction of dental alignment and establishment of a stable functional occlusion. The orthodontic objectives included maintaining the eruption pathway of the previously impacted teeth, correcting the existing malocclusion, achieving proper alignment and intercuspation and establishing a stable functional occlusion. Following marsupialization, spontaneous eruption of the mandibular canine and first premolar occurred without the need for orthodontic traction. Space was maintained for the erupted teeth with no additional surgical intervention or bone grafting required. No extractions were required during orthodontic treatment, as adequate space was available following spontaneous eruption of the mandibular canine and first premolar.

**Figure 7:**
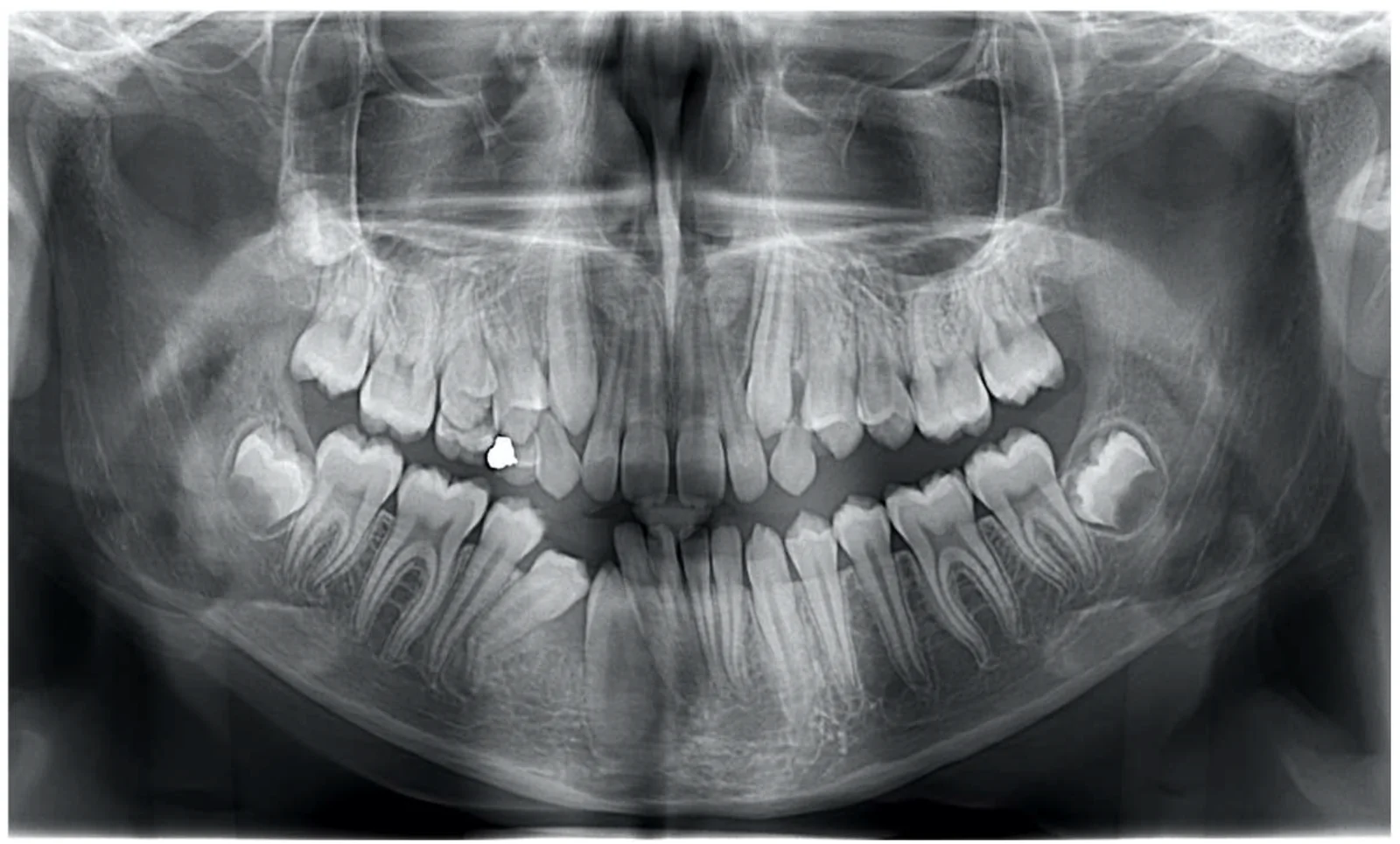
Panoramic radiograph 8.5 months post-surgery.

**Figure 8:**
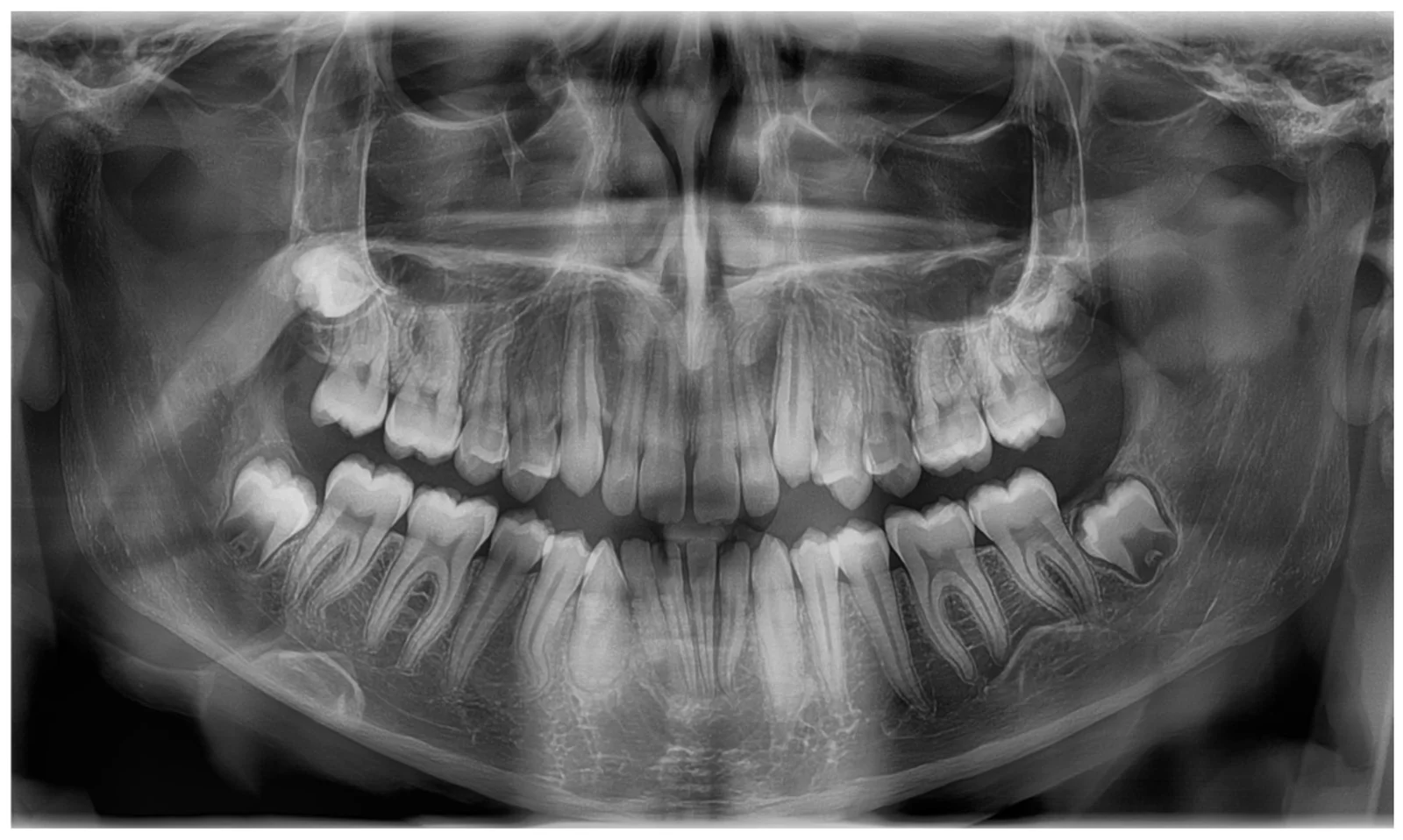
Panoramic radiograph 29 months post-surgery.

After 29 months of post-surgery follow-ups, when both impacted teeth had spontaneously erupted and the cyst and bone healing were considered satisfactory based on the radiographic examination (Figure 7 and Figure 8), the decision to initiate active orthodontic treatment was made. At the completion of the marsupialization phase, intraoral examination confirmed spontaneous eruption of both previously impacted teeth and favorable clinical conditions for initiation of orthodontic treatment.

**Figure 9:**
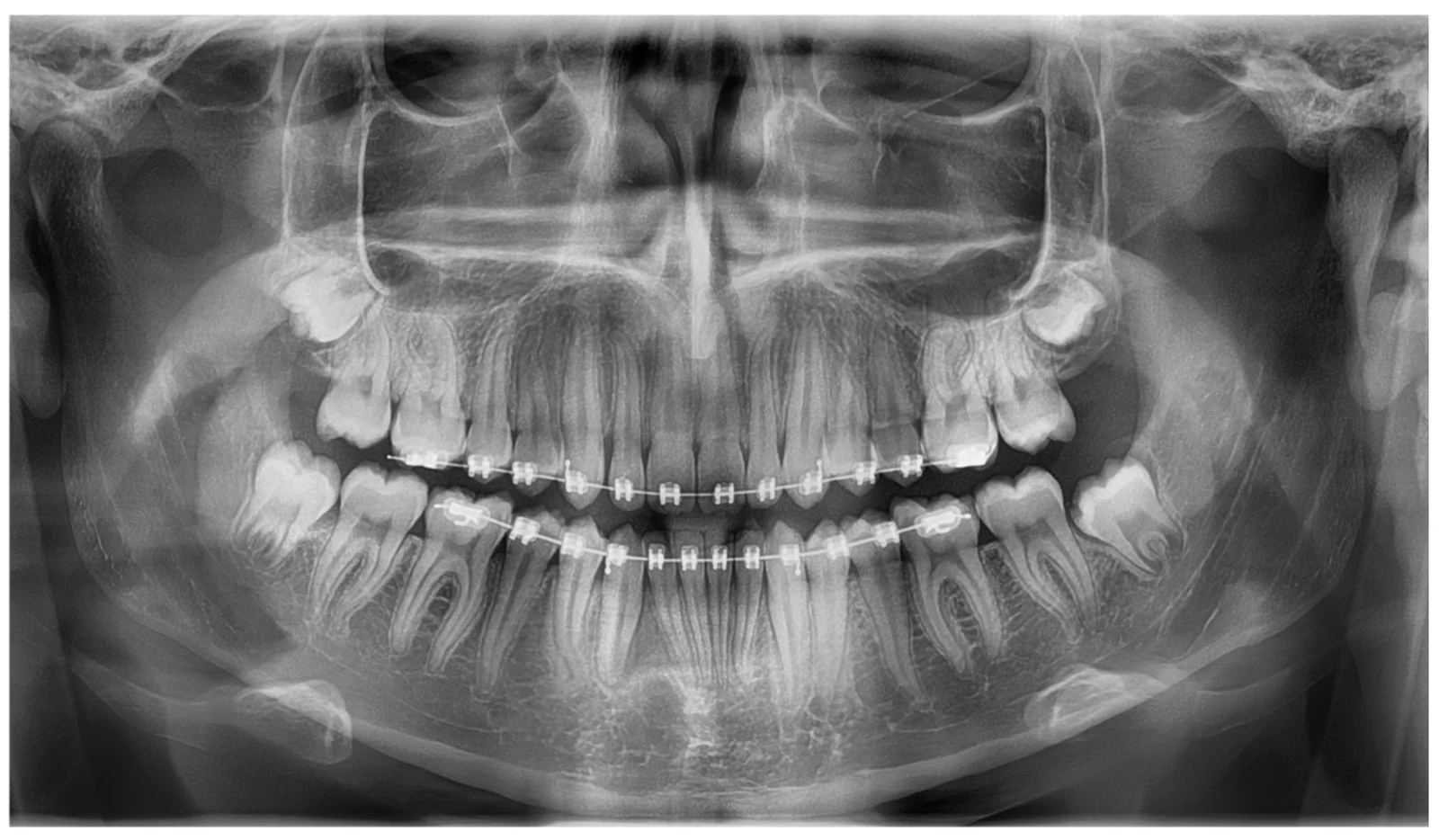
Pre-final panoramic radiograph.

Preadjusted edgewise 22-slot brackets were bonded in the upper arch. Treatment was initiated with 0.012-inch nickel–titanium archwires, which were gradually replaced with wires of increasing dimensions until leveling and alignment were achieved. Bonding in the mandibular arch was deferred for six months to allow for continued alveolar bone regeneration and the same sequence of archwires was followed. The patient attended monthly follow-up visits for appliance adjustment and activation. No additional anchorage reinforcement was required, as all planned tooth movements were accomplished using conventional preadjusted straight-wire appliance mechanics. The pre-final panoramic radiograph is depicted in Figure 9. A chronological overview of the diagnostic, surgical, orthodontic, and follow-up events is presented in Table 1.

### 2.5. Follow-Up and Treatment Outcome

**Figure 10:**
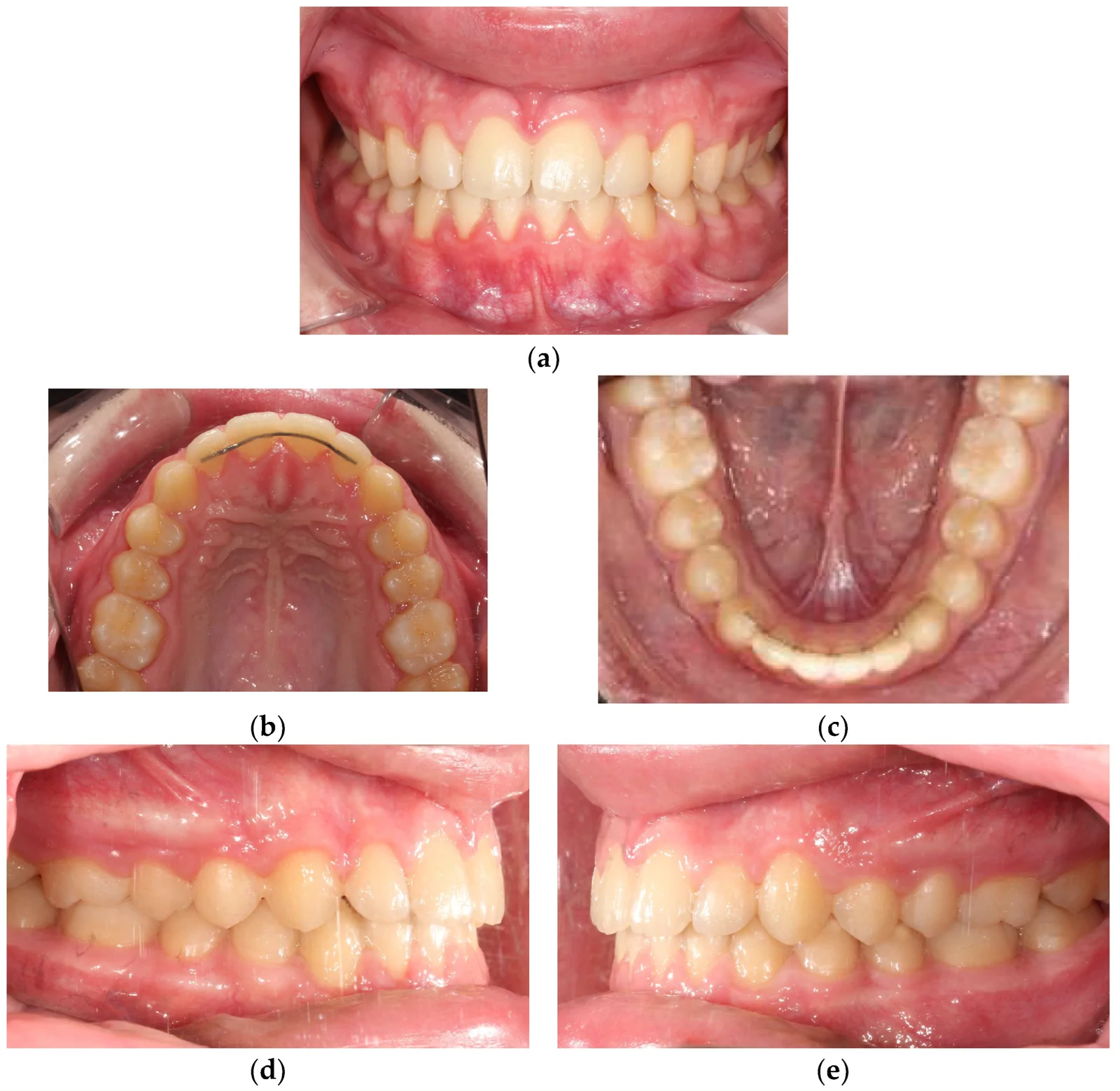
Final intraoral photographs obtained after completion of active orthodontic treatment: (**a**) frontal view, (**b**) maxillary occlusal view, (**c**) mandibular occlusal view, (**d**) right lateral view, and (**e**) left lateral view, demonstrating the final occlusal relationships and alignment achieved following interdisciplinary treatment.

**Figure 11:**
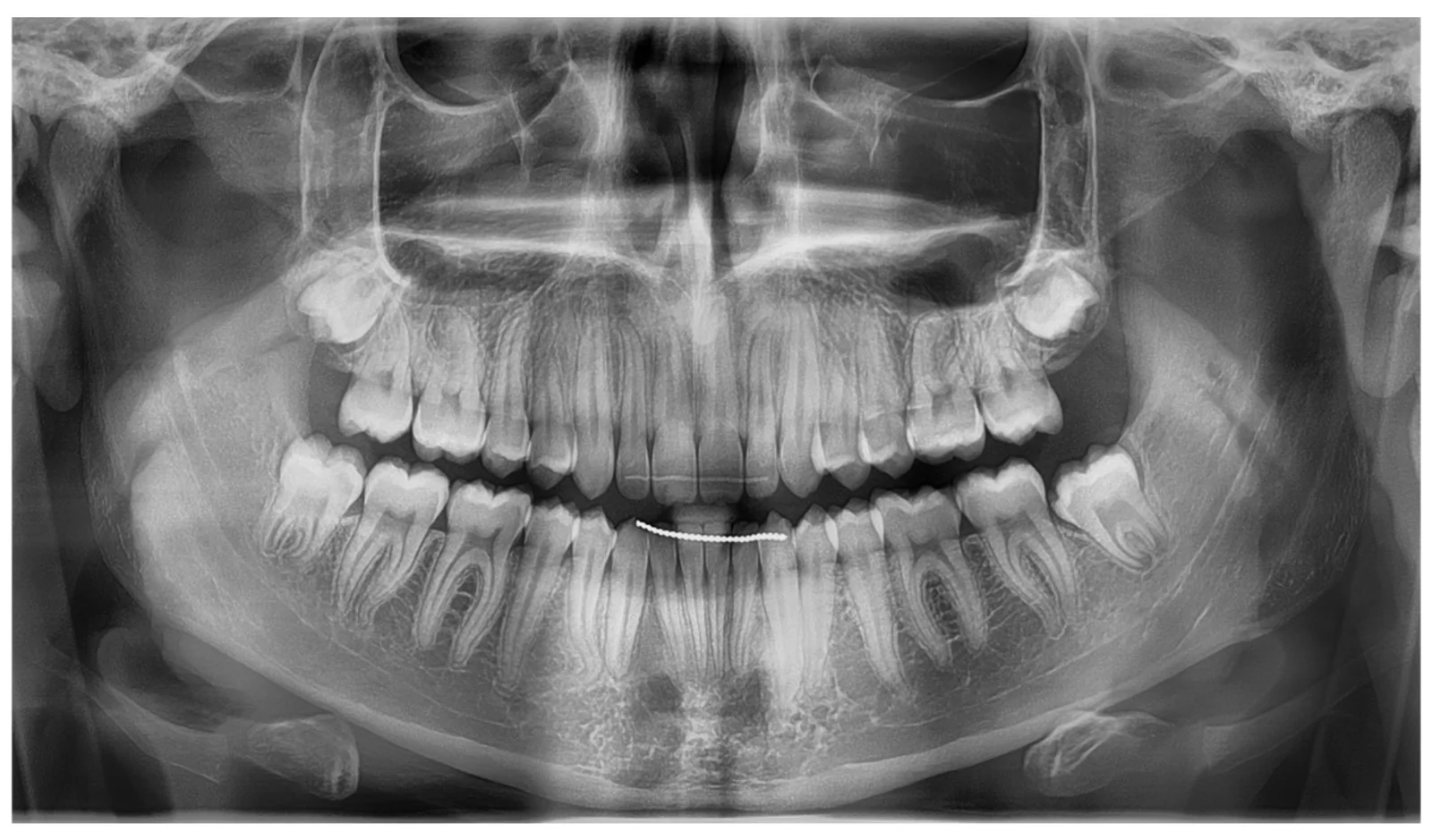
Final panoramic radiograph.

The overall treatment duration was 62 months, including 29 months of post-surgical monitoring and 33 months of orthodontic treatment. Need for bone grafting did not arise. At the completion of treatment, the periodontal condition of the affected and adjacent teeth was evaluated. Probing depths were within physiological limits, no pathological mobility was detected, gingival tissues appeared healthy, and no clinical attachment loss was observed. All involved and adjacent teeth retained their vitality as confirmed by cold vitality testing and electric pulp testing, at the completion of treatment. Fixed retainers were bonded to the lingual surfaces of the mandibular anterior teeth and the palatal surfaces of the maxillary anterior teeth, to preserve the achieved orthodontic alignment. Final treatment records are illustrated in Figure 10 and Figure 11. Semi-annual follow-up appointments were scheduled to ensure ongoing monitoring and maintenance. From the patient’s perspective, the treatment outcome was highly satisfactory. The patient reported satisfaction with the functional and aesthetic results and no treatment-related concerns or limitations during follow-up. At 18 months of clinical follow-up, the patient remained clinically stable, with no clinical signs suggestive of recurrence. The patient remains under follow-up, although no additional radiographic surveillance was performed.

### 2.6. Prognosis

The prognosis following successful marsupialization is highly favorable when the impacted tooth erupts naturally, accompanied by satisfactory bone regeneration and preserved tooth vitality. In such cases, the tooth is likely to function properly, with minimal risk of further complications. The surrounding bone typically heals well, providing a stable foundation for the tooth, and the overall dental and aesthetic outcomes are often excellent. This successful outcome suggests a low likelihood of relapse or the need for additional surgical interventions, promoting long-term dental health and function. In the present patient, the prognosis was favorable during the follow-up period. Marsupialization was followed by spontaneous eruption of the previously impacted permanent teeth, progressive radiographic evidence of bone regeneration, preservation of tooth vitality, and successful orthodontic alignment. These findings indicate a favorable clinical outcome in this individual case. However, continued clinical and radiographic surveillance remains important to monitor the stability of the erupted teeth, periodontal health, bone remodeling and any potential recurrence or late complications.

## 3. Discussion

Large dentigerous cysts, which are linked with two severely impacted teeth, as demonstrated in the presented clinical case, are rare occurrences. When such cases arise, they require interdisciplinary management, with orthodontists and oral surgeons having specific responsibilities in both the biological and technical aspects of the comprehensive treatment approach [15], while there is limited evidence available regarding the effectiveness of their treatment. In the present case, the decision to proceed with conservative management was based on several clinical considerations, including the patient’s age, the developmental stage of the involved permanent teeth, the potential for spontaneous eruption following decompression of the lesion and the radiographic characteristics suggesting a benign, well-defined cystic process.

The differential diagnosis of a well-defined pericoronal radiolucent lesion associated with an unerupted tooth includes an enlarged dental follicle, odontogenic keratocyst, unicystic ameloblastoma, adenomatoid odontogenic tumor, odontogenic myxoma and other odontogenic cystic lesions [16,17]. In the present case, the diagnosis of a dentigerous cyst was based on the characteristic clinical and radiographic findings, including the well-defined unilocular radiolucency associated with the crown of an unerupted permanent tooth, the displacement of the adjacent tooth, and the absence of features suggestive of an aggressive lesion. However, these findings are not pathognomonic, and histopathological examination remains necessary for definitive diagnosis and exclusion of other odontogenic entities. Therefore, the lesion in this report should be regarded as a clinicoradiographically diagnosed dentigerous cyst, with the absence of histopathological confirmation acknowledged as a limitation.

Successful marsupialization of a cyst with the preservation of impacted teeth is often considered superior to enucleation and extraction of permanent teeth due to its potential to preserve the patient’s natural dentition [8,9,10,11]. Retaining the teeth minimizes the need for future restorative interventions, such as implants or prosthetics, thereby supporting both the functional and aesthetic integrity of the dentition. From a clinical perspective, marsupialization also tends to involve less morbidity, promoting natural eruption of the impacted teeth while allowing for satisfactory cyst resolution and bone regeneration. However, the conservative approach may require prolonged monitoring and a longer overall treatment period compared with conventional surgical management. In the present case, the total treatment duration of 62 months exceeded the duration commonly reported for routine orthodontic treatment (mean 28.5 ± 10 months) [18], primarily due to the need for a 29-month post-marsupialization observation period to allow spontaneous eruption of the impacted teeth and satisfactory bone healing before initiating active orthodontic treatment, followed by 33 months of orthodontic correction.

In the presented case, a notable reduction in the size of the cystic lesion was observed, accompanied by improvements in both the quantity and trabecular structure of the mandibular bone. The interval between marsupialization and orthodontic treatment was a deliberate component of the treatment strategy rather than passive observation. Regular clinical and radiographic follow-up showed evidence of progressive lesion reduction, bone regeneration, and spontaneous eruption of the impacted teeth, allowing orthodontic treatment to be initiated only after favorable biological conditions had been achieved. Although this approach prolonged the overall treatment period, it aimed to avoid premature intervention and preserve the natural eruptive potential of the involved teeth. Additionally, the affected teeth underwent spontaneous eruption toward the occlusal plane, achieving successful alignment. Both the canine and the first premolar were subsequently supported by progressive radiographic osseous fill and a clinically healthy periodontium post-treatment, while remaining vital. Previous research indicates a high rate of spontaneous eruption for mandibular premolars, where the premolar remained vital, healthy, and well-supported by the periodontium upon reaching the occlusal plane [11,15,19,20,21]. Evidence suggests that marsupialization or decompression can facilitate spontaneous eruption of teeth associated with dentigerous cysts [22], with approximately 62% of affected premolars erupting without orthodontic traction. Younger patients and teeth with incomplete root formation appear to have the highest probability of successful spontaneous eruption [23].

Marsupialization and enucleation represent established treatment options for odontogenic cystic lesions, and the choice between them should be individualized according to lesion characteristics, pathological diagnosis, patient age, tooth development, anatomical relationships, and the potential risks and benefits of each approach. While marsupialization may preserve adjacent structures and allow continued eruption of involved teeth in selected young patients, enucleation may be preferred in cases where complete lesion removal is indicated or when clinical circumstances do not favor conservative management [7,8,14,15]. Therefore, the favorable outcome observed in the present case should not be interpreted as evidence of superiority of marsupialization over enucleation, but rather as an example of successful conservative management in a carefully selected patient.

From a patient’s perspective, marsupialization with the preservation of impacted teeth is often preferred because it is a less invasive procedure compared to enucleation, which involves the removal of both the cyst and potentially vital teeth. This minimally invasive approach reduces the risk of complications such as infection, nerve damage and excessive bleeding, leading to quicker recovery and less postoperative discomfort. Additionally, by maintaining the natural teeth, patients are spared the psychological and functional challenges associated with tooth loss, such as the need for implants, bridges or dentures. The preservation of natural teeth also helps to maintain proper dental alignment and function, which is especially important for younger patients whose teeth and jaw are still developing. From an aesthetic standpoint, keeping the natural teeth ensures a more aesthetically pleasing outcome, avoiding the need for future cosmetic dental work. Ultimately, this approach supports the patient’s long-term dental health, minimizes future interventions and provides a more positive overall treatment experience. Moreover, this patient-centered approach aligns with principles of respect for the patient’s preferences, values and autonomy, ensuring that treatment decisions are made collaboratively, with the patient’s best interests at the forefront of care [24].

The clinical relevance of this case does not rely solely on the spontaneous eruption of impacted teeth, which has previously been described following conservative management of dentigerous cysts. Rather, the value of this report lies in the combination of a large mandibular lesion involving two permanent teeth, a prolonged follow-up period demonstrating progressive healing and eruption, and the subsequent integration of orthodontic treatment after biological recovery had occurred. This case highlights a staged interdisciplinary approach in which surgical intervention was limited initially to lesion management, allowing the patient’s developmental potential to contribute to tooth eruption and bone regeneration before orthodontic correction.

The present report has several limitations that should be considered when interpreting the findings. First, as a single-case report, the observations cannot be generalized to all patients presenting with similar lesions, and further studies with larger samples and longer follow-up are required. Second, the absence of histopathological confirmation represents an important limitation, as the definitive diagnosis of odontogenic cystic lesions requires microscopic evaluation. Although the lesion demonstrated the characteristic clinical and radiographic features of a dentigerous cyst, including a well-circumscribed pericoronal radiolucency associated with an unerupted tooth, these findings alone cannot establish a definitive diagnosis [25]. Other odontogenic cysts and tumors may present with similar imaging characteristics. Consequently, the diagnosis in this report should be regarded as presumptive and based on the overall clinicoradiographic presentation. Nevertheless, the favorable long-term clinical course, spontaneous eruption of both impacted teeth, progressive osseous regeneration, and absence of recurrence are highly indicative of the benign nature of the lesion and support the appropriateness of the conservative management adopted in this patient, while not replacing the diagnostic value of histopathological examination. Additionally, bone regeneration was assessed qualitatively through sequential conventional radiographic examinations rather than through quantitative measurements or CBCT-based volumetric analysis. Although the radiographic findings demonstrated progressive osseous healing, objective measurements would provide a more precise evaluation of the extent of bone regeneration. Finally, treatment decisions should be individualized and based on multiple factors, including patient age, developmental stage, expectations, compliance, lesion characteristics, and the potential for preservation and eruption of the involved teeth. Finally, no additional radiographic follow-up after appliance removal was available. Therefore, long-term assessment of lesion recurrence or continued bone remodeling beyond the treatment period remains limited.

## 4. Conclusions

The marsupialization of the cyst led to the spontaneous eruption of the two involved, impacted teeth, accompanied by bony fill-in to normal interproximal alveolar height, improved quantity and trabeculation of the mandibular bone, while the subsequent orthodontic treatment resulted in the successful alignment of the teeth. In this adolescent patient, this conservative approach was associated with favorable clinical and radiographic outcomes. However, as a single case report without histopathological confirmation, these findings should be interpreted with caution and cannot be generalized to all patients with similar lesions. Although a biologically driven treatment philosophy should guide decision-making, successful interdisciplinary management requires the integration of appropriate technical interventions when indicated. In this context, surgical and orthodontic procedures should complement, rather than replace, the patient’s inherent biological potential for healing, bone regeneration, and tooth eruption. This approach emphasizes the natural healing capacity and developmental potential of the patient, particularly in pediatric and young adult cases. Instead of immediately adopting more invasive or complex interventions, this strategy prioritizes minimally invasive procedures when appropriate, supporting physiological processes such as spontaneous tooth eruption and bone regeneration while aiming to optimize long-term functional and esthetic outcomes. It also promotes individualized care by considering factors such as patient age, developmental stage, clinical characteristics, and cooperation, thereby facilitating a more efficient and less traumatic treatment process.

## Figures and Tables

**Table 1:** Chronological timeline of clinical, surgical, orthodontic, and follow-up events.

Time Point	Clinical Event/Intervention	Clinical and Radiographic Findings
Initial presentation (Age 14 years)	Referral to orthodontic clinic by general dentist for evaluation following extraction of a non-vital primary mandibular molar	Extraoral examination revealed a well-balanced, relatively symmetrical face with a straight profile. Intraoral examination showed mixed, spaced dentition with bilateral Class I molar relationships.
Initial radiographic assessment	Periapical radiograph obtained from referring dentist	Radiolucent lesion identified between the apex of the extracted primary molar and the cemento-enamel junction of the permanent successor premolar. Patient referred for further imaging.
Diagnostic imaging	Panoramic radiograph and localized CBCT performed	Large, well-defined radiolucent lesion identified in the mandibular body. Lesion associated with the crown of an unerupted first premolar and displacement of the adjacent permanent canine. Two impacted permanent teeth were identified (mandibular canine and first premolar).
Initial treatment planning	Multidisciplinary evaluation	Conservative management selected with the aim of resolving the lesion, preserving permanent teeth, promoting spontaneous eruption and allowing subsequent orthodontic correction. Lesion described as a clinicoradiographic dentigerous cyst due to the absence of histopathological confirmation.
Surgical phase (Month 0)	Marsupialization performed under local anesthesia by an oral surgeon	Preoperative aspiration was performed, yielding clear, straw-colored fluid. Surgical drainage was achieved through the extraction socket of the deciduous molar. A portion of the cystic lining was excised, the epithelial lining was sutured to the adjacent oral mucosa and an acrylic obturator was placed to maintain patency of the opening.
Early postoperative period	Obturator maintenance and irrigation protocol	Patient performed twice-daily irrigation with normal saline. The obturator was maintained for 6 months and was periodically adjusted according to healing progress and eruption changes. No treatment-related complications were observed.
Postoperative monitoring period	Regular clinical and radiographic follow-up	Serial radiographs demonstrated progressive reduction of the radiolucent lesion and favorable bone healing. The patient demonstrated good compliance with the irrigation protocol.
Completion of marsupialization phase	Transition to orthodontic treatment planning	Marsupialization was considered complete after clinical resolution, sustained reduction in the radiolucent area, progressive radiographic bone healing and spontaneous eruption of the associated impacted teeth.
29 months after marsupialization	Initiation of active orthodontic treatment in the upper arch	Both impacted permanent teeth had spontaneously erupted. Cyst resolution and satisfactory bone healing were confirmed radiographically. Preadjusted edgewise 22-slot brackets were bonded in the maxillary arch. Mandibular bonding was delayed for six months to allow further alveolar bone maturation. Treatment started with 0.012-inch nickel–titanium archwires followed by progressive wire sequence.
Orthodontic treatment phase (33 months)	Monthly orthodontic appointments for activation and adjustment	Progressive leveling and alignment achieved. No bone grafting was required. All involved and adjacent teeth maintained vitality.
Completion of active treatment (62 months after initial presentation)	Orthodontic treatment completed	Final treatment records obtained. Fixed retainers bonded to maxillary and mandibular anterior teeth to maintain alignment.
Post-treatment follow-up	Semi-annual monitoring	Continued clinical monitoring for stability and maintenance. Patient reported satisfactory functional and aesthetic outcomes.

## Data Availability

Data supporting the findings of this case report are not publicly available due to patient privacy and ethical restrictions. All relevant information is included within the article.
